# Cardiovascular Fitness and Potential Risk of Work-Related Injuries in Manual Therapy Students: A Cross-Sectional Study

**DOI:** 10.7759/cureus.84053

**Published:** 2025-05-13

**Authors:** Qian Zhang, Zhicheng Yao, Yifei Ma, Jeng-Sheng Yang

**Affiliations:** 1 Department of Research, Nuo De Fu Sports, Beijing, CHN; 2 College of Pharmacy, Tianjin University of Traditional Chinese Medicine, Tianjin, CHN

**Keywords:** cardiovascular rehabilitation, medical student health, occupational lung diseases, occupational risk, vo2 max

## Abstract

Background

Chiropractic and manual therapy professions are considered physically demanding and tend to be associated with high rates of work-related injuries. The reported injuries particularly affect the upper extremities and lower back. Despite extensive research on physical therapists, there are limited studies on chiropractors. This study aims to evaluate the cardiovascular fitness of manual therapy students to evaluate their risk of potential work-related injuries.

Methods

This cross-sectional study recruited 40 (17 to 26-year-olds) healthy male and female manual therapy students from Beijing Sports University. Participants performed the single-stage treadmill walking test to estimate their maximal oxygen uptake (VO₂ max). Body mass index (BMI) was calculated, and data were analyzed to compare physical and physiological variables between the gender and weight categories.

Results

Male students had a higher mean VO₂ max (51.23 ± 6.13 mL/min/kg) compared to female students (39.62 ± 3.81 mL/min/kg). Normal weight individuals had a slightly lower mean VO₂ max (47.40 ± 8.30 mL/min/kg) than overweight individuals (47.64 ± 6.14 mL/min/kg). The study also highlighted significant differences in height, weight, and BMI between genders.

Conclusions

The study indicates that manual therapy students have high VO₂ max values, suggesting good cardiovascular fitness, which is essential for their physically demanding careers. However, the higher VO₂ max in males and the minor difference between normal and overweight individuals suggest the need for tailored fitness programs. Preventive strategies to enhance physical resilience and reduce the risk of work-related injuries are imperative for students pursuing careers in chiropractic and manual therapy. Future research should focus on larger, more diverse samples and direct VO₂ max measurements for a more accurate evaluation of cardiovascular fitness and its influence on occupational performance.

## Introduction

The development of modern physiology and kinesiology has heightened the awareness of personal health and the significance of living a healthy lifestyle. This significance could be directly applied to professionals, such as chiropractic and manual therapy clinicians, who encounter high physical demands. A previous survey of 397 randomly selected Doctors of Chiropractic Medicine in the US found that 159 chiropractors reported 252 work-related injuries. Among all the injured body parts, upper extremity and low back injuries were at the top of the list [1]. Another study that involved 62 chiropractors reported a lifetime prevalence of work-related musculoskeletal injuries of 69% [2]. Regarding those injuries, 50% were targeted at the upper extremities and 28.3% targeted at the lower back. These findings demonstrated the strenuous nature of chiropractic and manual therapy professions, which demand a high level of physical fitness [2].

A great variety of studies have examined work-related injuries among physical therapists. Results showed a widespread prevalence and significant impact of musculoskeletal disorders within the profession. One systematic review analyzed 26 studies out of 722 articles, revealing a high global prevalence rate among physiotherapists, particularly in the lower back, neck, and shoulder regions [3]. Also, research conducted by Campo et al. demonstrated that 55% of physical therapists in the United States have experienced work-related injuries [4]. Researcher Alrowayeh et al. found an intriguingly higher prevalence of 78% work-related musculoskeletal injury rate among physical therapists in Kuwait [5]. Furthermore, Vieira et al. identified that 65% of Brazilian physical therapists reported work-related musculoskeletal pain [6].

Compared to the physical therapy profession, there was less high-quality research on work-related injuries for chiropractic clinicians. Among the few studies, a 2018 cross-sectional study showed that 216 chiropractors reported at least one work-related physical injury or overuse complaint in the previous year [7]. Interestingly, chiropractors with more than five years of practice reported significantly fewer work-related acute injuries and overuse complaints as compared to those with less than five years of practice [1]. Overall, a shortage of significant studies focusing on occupational injuries among chiropractors highlights a considerable gap in understanding the occupational health challenges they face compared to physical therapists.

Based on the existing chiropractic work-related injury reports and the physically demanding nature of chiropractic work, further research should be done to address the mechanism of how this happens and what could be done to prevent and alleviate this challenge. To start with addressing this gap, further studies should focus on the fitness levels of chiropractors. Based on the results, feasible preventive strategies should be developed to enhance their physical resilience and minimize the incidence of work-related injuries.

Physical fitness can be defined as the capacity to perform physical tasks effectively under diverse environmental conditions. Based on the definition, cardiorespiratory fitness has been recognized as a critical indicator of future health and overall functional capacity [8]. Research conducted by Tarp et al. has demonstrated a linear correlation between cardiorespiratory fitness and the risk of developing cardiovascular disease and type 2 diabetes within the general population [9]. Nevertheless, individuals with elevated levels of cardiorespiratory fitness exhibit a reduced risk of these conditions [10]. Furthermore, A study has quantified the relationship between cardiovascular disease and cardiorespiratory fitness, providing a clear ratio that highlights the importance of maintaining high fitness levels [11]. A study implemented by Ras et al. stated that physical fitness, including aerobic capacity, plays a protective role in occupational injury by improving endurance during repetitive physical tasks and reducing fatigue-related movement errors [12].

Low cardiorespiratory fitness may contribute to poor fatigue resistance, which easily leads to muscle fatigue [13]. Typically, muscular fatigue is associated with a decline in postural stability [14]. Good postural stability can indirectly reduce the mechanical loading on regions like the spine and shoulders during repetitive or awkward manual tasks [15,16].

Within the lab-based assessment, the most widely used method to measure cardiorespiratory fitness level is the maximal oxygen uptake(VO₂ max) test [17]. The VO₂ max test is the standard approach for both professional athletes and the general population. The VO₂ max is considered an essential indicator of an individual’s cardiovascular function. VO₂ max is influenced by two main factors: maximal cardiac output and arteriovenous oxygen difference. VO₂ max across different populations shows significant differences due to different cardiac output abilities. As a result, VO₂ max is strictly associated with the performance of the heart in individuals. The test mainly aimed to accurately reflect the individual performance of the VO₂ max test with fully achieving maximal physiological limits. What’s more, the study stated that VO₂ max and grip strength are reversely associated with the risk of musculoskeletal pain among construction and healthcare workers, suggesting the benefit of good physical fitness in occupational health [18].

This study focuses on evaluating maximal oxygen uptake in young adult male and female manual therapy students who are willing to pursue a career in the chiropractic profession or physical therapy and other manual therapy professions at Beijing Sports University. The goal is to measure the baseline cardiorespiratory fitness and analyze their physical fitness levels to assess their risk of potential work-related injuries.

## Materials and methods

Inclusion and exclusion criteria

The recruitment criteria included: 1. Healthy male and female subjects without any preexisting cardiovascular diseases or any acute or chronic illnesses or lower extremities injuries; 2. Non-smokers and individuals who do not consume alcohol daily; Students intending to pursue careers as chiropractors, physical therapists, or any other type of manual therapists after graduation.

Participants

This study selected 40 students from 87 target participants. Data were collected from 27 male students and 13 female students.

The participants’ ages ranged from 17 to 26 years. Based on the exclusion criteria, participants who have pre-existing cardiovascular conditions were excluded. Those cardiovascular conditions included arrhythmias, coronary artery disease, hypertension, and a history of heart attacks. The exclusion helped reduce health risks and kept the study group consistent. Participants with a lower extremity sprain or strain were also excluded. This prevented further injury and ensured similar physical conditions among participants.

All participants were informed of all relevant instructions and potential risks before signing the consent form. The course instructor approved the study protocol prior to the start of the research. For confidentiality, all personal information was removed.

Equipment

Without maximal exertion, the single-stage treading walking test is commonly applied to estimate VO₂ max. This protocol is suitable for moderate-sized groups and minimizes equipment requirements: only a treadmill and an HR monitor.

To ensure inter-rater reliability and standardized equipment calibration, all VO₂ max tests were conducted with one treadmill and one HR monitor. The same researcher administered all testing sessions throughout the study to ensure consistency of the test. Prior to the test beginning, equipment was calibrated following the manufacturer’s recommendation.

Procedure

The test was conducted from October 7, 2024, to October 11, 2024.

Before beginning the test, researchers need to inform all participants of the test’s primary objective, experiment procedures, and measurements needed to be assessed. For calculating the predicted maximal heart rate, we utilize the formula: 220 - age. We also calculated the 50% and 70% of each subject's age-predicted maximal heart rate (HR max). Additionally, subjects were instructed to warm up for 4 minutes at a 0% grade and a walking speed that brings the HR between 50% and 70% of their maximum. Then, the subject was instructed to walk at a speed range between 5.4. and 6.4 km*hr-1. After completing the warmup section, the subject is required to maintain the same speed for an extra 4 minutes at a grade of 5% and record the steady state HR at the end of 4 minutes. If there is > 5 b*min-1 difference, the subject should be instructed to continue the test for an extra minute. The following equation should be used to estimate in ml∙kg-1∙min-1 [19].

VO2 max

= 15.1 + (21.8 x speed in miles per hour)

- 0.327 *final HR

- 0.263 *speed *age

+ 0.00504 *HR *age

+ 5.98 *gender (0= females, 1= males)

Body mass index (BMI) was calculated as body weight in kilograms divided by the square of body height in meters. Normal weight was defined as BMI ranging from 18.5 to 24.9, underweight as BMI less than 18.5, overweight as BMI from 25 to 29.9, and obesity as BMI 30 or greater [20].

## Results

As mentioned in Table 1, the mean height of male participants (176.12 ± 6.57 cm) was notably higher than that of female participants (163.94 ± 5.88 cm). Similarly, the mean weight of males was 74.70 ± 12.72 kg as compared to 62.15 ± 7.56 kg for females. Regarding BMI, the mean BMI of males as 24.00 ± 3.42 kg/㎡, slightly higher than the female mean BMI of 23.10 ± 2.24 kg/㎡. The VO₂ max, a critical measure of aerobic capacity, showed a substantial difference between genders. The mean VO₂ max among males was 51.23 ± 6.13 mL/min/kg, significantly higher than the female mean of 39.62 ± 3.81 mL/min/kg (Table 1).

**Table 1:** Comparison of physical and physiological variables between male and female participants VO₂ max: maximal oxygen uptake

Variables	Male (mean ± sd)	Female (mean ± sd)
Age (Years)	19.96±2.47	19.85±2.27
Height (cm)	176.12±6.57	163.94±5.88
Weight (kg)	74.70±12.72	62.15±7.56
BMI (kg/㎡)	24.00±3.42	23.10±2.24
VO₂ max (mL/min/kg)	51.23±6.13	39.62±3.81

Table 2 showed that only 10 (25%) subjects fell into the category of normal weight according to BMI, and 30 (75%) subjects fell into the overweight category. When combining all subjects, both male and female, the VO₂ max for normal weight individuals was 47.40 ± 8.30 mL/min/kg, while for overweight individuals, it was slightly higher at 47.64 ± 6.14 mL/min/kg. A further breakdown by gender showed that males in the normal weight group had higher VO₂ max (51.98 ± 6.41 mL/min/kg) than overweight males (49.47 ± 5.40 mL/min/kg). For females, normal weight individuals had a VO₂ max of 39.49 ± 4.14 mL/min/kg, whereas overweight females had a slightly higher VO₂ max of 40.33 ± 1.20 mL/min/kg (Table 2).

**Table 2:** The predicted VO₂ max between normal weight and overweight individuals across different groups VO₂ max: maximal oxygen uptake

Variables	Normal weight (mean±sd)	Overweight (mean±sd)
All subjects (male + female)	10	30
All subjects (male + female) VO₂ max (mL/min/kg)	47.40±8.30	47.64±6.14
Male VO₂ max (mL/min/kg)	51.98±6.41	49.47±5.40
Female VO₂ max (mL/min/kg)	39.49±4.14	40.33±1.20

## Discussion

The increasing incidence of occupational injuries among manual therapy professionals, such as chiropractors and physical therapists, shows that practitioners require a high level of physical fitness. The purpose of this study was to examine the physical fitness level of students who were preparing for future careers in these fields, especially focused on their cardiorespiratory fitness, as measured by VO₂ max. Our findings raised awareness of the physical demands and potential work-related injuries inherent to those professions.

Our results suggest that subjects who were pursuing careers as manual therapists exhibit higher VO₂ max values as compared to other populations. Previous research conducted with 57 medical students in India reported that an average VO₂ max among male students was 45.66 ± 8.9 ml·kg-1·min-1 and 37.85 ± 4.3 ml·kg-1·min-1 among female students [21]. On the contrary, our results showed higher VO₂ max values for both male and female students. This discrepancy may be because of manual therapy students who possess sports rehabilitation courses in their curriculum that lead them to engage in developing exercise routines.

These findings also raise awareness of the possible risk for work-related injuries (WRIs) in the manual therapy profession. Research conducted on physical therapists (PTs) showed considerable prevalence of WRIs, musculoskeletal pain, and musculoskeletal disorders (WMSDs). Furthermore, a prospective cohort study by Campo et al. found a one-year incidence of 20.7% for moderate to major WMSDs in any body region among a randomly selected national sample of 882 PTs [4]. The study defined WMSDs as pain with a minimal severity of 4/10, experienced at least monthly or persisting for more than a week [22]. Besides physical therapists, other manual therapists, like chiropractors, experienced high incidences of WRIs as well. According to the study by Cote et al., 79% of chiropractors experienced WRMSDs, with the low back, neck, and shoulders being the most commonly affected areas [23]. Similarly, as reported by Bork et al., 91% of physical therapists suffered from WRMSDs, highlighting the physical strain experienced by professionals in these fields [24]. These findings emphasize the necessity for students who are entering this profession should be aware of these risks and utilize a well-structured training program to maintain a good physical fitness level.

The well-maintained VO₂ max values observed in our study's manual therapy students suggest better overall cardiorespiratory fitness. This good physical condition could be beneficial in mitigating some of the physical demands of their future profession. Higher cardiorespiratory fitness demonstrates better fatigue resistance, enhanced recovery, and improved muscular endurance, all of which may contribute to reducing biomechanical strain and compensatory movement patterns during occupational tasks [13]. With the potential benefit of high-level cardiorespiratory fitness, it is imperative to establish fitness programs that improve physical resilience and lower the risk of musculoskeletal disorders.

Our data showed VO₂ max values among both sexes classified within healthy ranges. However, data of male students generally had higher VO₂ max levels as compared to female students (51.23±6.13 mL/min/kg vs. 39.62±3.81 mL/min/kg). This discrepancy aligns with general physiological differences between genders and highlights the need for tailored fitness programs that consider these differences to enhance physical resilience and reduce injury risk.

What’s more, the study represents an overview of the baseline cardiorespiratory fitness level of manual therapy students. Knowing the cardiorespiratory fitness levels of students and the physical demands of clinical practice can help identify a shortcoming in fitness levels. This understanding is meaningful for curriculum design, as it supports the targeted physical training to enhance student fitness and reduce occupational risks.

Furthermore, the data revealed that females tended to have higher end heart rates compared to males during submaximal exercise, indicating weaker cardiorespiratory fitness levels. This supports the notion that engaging in regular exercise can significantly enhance cardiorespiratory fitness, as demonstrated by the positive effect of low-volume sprint interval training on aerobic power and endurance [25,26].

It has to be acknowledged that there are several limitations within our study. First of all, our sample size was relatively small (n=40), which may not fully represent the broader population of chiropractic and manual therapy students. Also, our study was conducted at a single institution, and it could create a potential limitation of the generalizability of the findings. Future research should adopt larger and more diverse sample groups across multiple institutions so that the external validity of the results can be enhanced. Although the cardiorespiratory data from the India research have been included, the lack of a control group in this study remains an important limitation. Future studies should utilize a matched control group to not only allow for precise comparisons but also improve the research quality and generalizability of the findings.

Moreover, self-reported physical activity levels and potential biases in reporting cannot be ruled out, which might have affected the accuracy of the physical fitness assessments. More detailed information-gathering on individual lifestyles and exercise preferences could provide more comprehensive insights.

Research conducted with 605 male firefighters reported a mean VO₂ max (43.3 ± 9.8 ml·kg-1·min-1) [27]. The discrepancy of the VO₂ max values between current findings and college students raises concerns about the accuracy of the submaximal treadmill walking test used in this study. Although the test is practical and less strenuous, it may not provide measurements as precise as those obtained from maximal exercise tests. The reliance on calculations rather than direct measurement introduces a risk of overestimation. For more accurate results, future studies should consider using open-circuit spirometry to measure VO₂ max directly by assessing respiratory volume and exhaled levels of oxygen and carbon dioxide [28].

## Conclusions

This study established a baseline test for assessing the cardiovascular fitness levels of manual therapy students, highlighting the critical role of physical fitness for those pursuing careers in chiropractic and manual therapy. The observed cardiorespiratory fitness levels may be relevant to work-related injuries in these careers, suggesting an optional connection between cardiorespiratory fitness levels and occupational risk. Given the high incidence of work-related injuries among professionals in these fields, these findings highlight the value of building preventive strategies and fitness programs. Future research should aim to address the identified limitations and build upon these findings to develop comprehensive guidelines for physical fitness in manual therapy professions. Such guidelines will improve practitioners' well-being and may potentially contribute to better patient care outcomes in the future.
